# Association of Glycated Hemoglobin With Acute Ischemic Stroke in a Tertiary Care Center in a Tribal Region of Jharkhand

**DOI:** 10.7759/cureus.58797

**Published:** 2024-04-23

**Authors:** Shimpy Priya, Vikas Mardi, Siddharth Kapoor, Abhay Kumar, Usha Saroj, Ajit Dungdung, Raunak Rishu

**Affiliations:** 1 General Medicine, Rajendra Institute of Medical Sciences, Ranchi, IND; 2 Blood Bank, Rajendra Institute of Medical Sciences, Ranchi, IND; 3 Internal Medicine, Rajendra Institute of Medical Sciences, Ranchi, IND; 4 Radiology, Jawaharlal Nehru Medical College, Aligarh, IND

**Keywords:** ischemic stroke, case control studies, jharkhand, random blood sugar (rbs), glycated hemoglobin (hba1c)

## Abstract

Background: Increased glycated hemoglobin (HbA1c) levels have shown an association with an increased risk of stroke in patients admitted to a tertiary care center in Jharkhand.

Objectives: To find out and estimate the risk of acute ischemic stroke in patients with increased HbA1c levels compared with controls.

Methods: This observational case-control study was conducted on patients admitted to the department of general medicine at a tertiary care center in Ranchi from June 2021 to November 2022. The patients included in this study were those aged 18 years or older and who were clinically and radiologically diagnosed with acute ischemic stroke. Only patients with a first episode of stroke were included, and patients with hemorrhagic stroke or transient ischemic attack were excluded from this study. An equal number of control participants were also included. Ion exchange high-performance liquid chromatography was used to perform the HbA1c tests. The same method was used to measure HbA1c levels in the controls. All findings were recorded in a Microsoft Excel sheet (Microsoft Corporation, Redmond, WA), and the data were analyzed using SPSS version 22.0 software (IBM Corp., Armonk, NY). After performing a descriptive statistical analysis, the findings were classified over a range of values and described accordingly. For each variable, an independent t-test was performed to compare the cases with the controls. A multivariable logistic regression analysis was used to choose the appropriate potential factors to determine the association in the multivariable analysis.

Results: A total of 185 cases and 185 controls were included. The mean age of the cases with ischemic stroke was 63.77 ± 10.312, and that of the controls was 53.18 ± 11.35 (p < 0.01). The mean HbA1c level in the patients of acute ischemic stroke was 6.97 ± 1.84, and that of the controls was 5.99 ± 1.69 (p < 0.01). The mean random blood sugar (RBS) value in the ischemic stroke cases was 170.21 ± 84.16, and that of the controls was 150.03 ± 82.25 (p = 0.02). To compare the factors that were determined to be statistically significant between ischemic stroke cases and controls, a multivariable logistic regression analysis was performed. The HbA1c p-value was 0.01, the odds ratio (OR) was 1.280, and the 95% CI was 1.11-1.48. The other variables apart from HbA1c that were statistically significant between the ischemic stroke cases and the controls were age (p < 0.01, OR: 1.280, 95% CI: 1.06-1.11), hypertension (p = 0.618, OR: 1.130, 95% CI: 0.70-1.83), and high-density lipoprotein (p = 0.055, OR: 0.975, 95% CI: 0.95-1.00). When other cofounders were considered, it was concluded that with a 1% increase in HbA1c, the risk of stroke increases by 28% (OR: 1.28, 95% CI: 1.11-1.48). To compare the variables that were determined to be statistically significant between the control and ischemic stroke case groups, a multivariable logistic regression was used. The area under the receiver operating characteristic curve for HbA1c was 0.773 and RBS was 0.600.

Conclusion: This study shows that higher HbA1c levels in patients increase the risk of ischemic stroke. This study brings to light the need to screen the population periodically for diabetes by routinely testing for Hba1c in those who are at high risk of diabetes. Stroke risk can be reduced with early management and intervention. This study also concludes that HbA1c is a better predictor for assessing the risk of ischemic stroke than RBS levels.

## Introduction

The abrupt onset of a focal neurological deficit caused by a focal vascular source is known as a stroke. Cerebral ischemia results from a decrease in blood flow. If ischemia persists for long enough to cause irreversible damage, then an infarction occurs. The second most common cause of mortality across the globe is stroke, accounting for 6.2 million deaths in 2015, an increase of 8,30,000 since 2000 [1]. In the last few decades, India has experienced substantial changes in its population, economy, and epidemiology. These have led to an aged population and an increase in life expectancy. There are very few accurate estimates of stroke morbidity and mortality available in India [2].

The primary cause of most ischemic strokes is arteriosclerotic plaques, which can cause a blood clot by activating the blood's clotting mechanism and, therefore, obstruct the blood supply to the brain, resulting in an abrupt loss of neurological function in a specific location [3]. Hypertension, diabetes, atherosclerosis, dyslipidemia, smoking, brain aneurysms, and arteriovenous malformations are some of the major risk factors for stroke [4].

Several factors related to the increased risk of stroke, such as atherosclerosis, increased carotid artery thickness, cardiomyocyte dysfunction, coronary vascular disease, and atrial fibrillation, have been related to chronic hyperglycemia [5]. Chronic hyperglycemia damages small and medium-sized blood vessels as well as the muscles in large and medium-sized arteries, which is the cause of the morbidity linked to long-term diabetes. In red blood cells, non-enzymatic covalent attachment of glucose moieties to hemoglobin results in the formation of glycated hemoglobin (HbA1c). This process offers some degree of glycemic regulation during a red cell's 120-day lifecycle [6].

An average blood glucose level of 135 mg/dL was associated with an HbA1c of 6% in the diabetes control and complication trial, and since then, HbA1c has been the main indicator of diabetes [7]. HbA1c is used to identify diabetes and prediabetes and assess how well a diabetic's blood glucose is being managed in the long term [8]. HbA1c is not affected by day-to-day variations and is recommended to be maintained below 7% in diabetic patients [6]. A first-time increased HbA1c level is associated with poor functional outcomes in patients admitted for acute ischemic stroke [9]. Individuals may suffer from loss of eyesight, speech difficulties, motor and/or sensory deficiencies in one or more limbs, and trouble doing daily tasks [10]. A physical examination is the main basis for diagnosis, which can be supported by imaging tests, such as an MRI or CT scan. While ischemia may not always be ruled out by a CT scan, bleeding may be excluded [11].

## Materials and methods

Observational case-control research was conducted at the Department of Medicine, Rajendra Institute of Medical Sciences (RIMS), Ranchi, from June 2021 to November 2022. A total of 185 patients who were identified as having an acute ischemic stroke both clinically and by imaging were admitted to the Department of Medicine, RIMS, Ranchi. An equal number of control patients were also collected from the same hospital. The sample size was initially determined using the formula: SS = z2p(1-p)/e2, where SS = sample size, z = statistic for the level of confidence, p = prevalence, and e = precision.

Using a statistical confidence level (z) of 1.96 (taking a confidence interval of 95%), a prevalence (p) of 0.5 (there is no published data regarding the prevalence of acute ischemic stroke in the state of Jharkhand, so it is assumed to be 50%), and a precision (e) of 0.05, the sample size (ss) is 384. Using the formula for a finite population size (N), the formula for the new sample size (SS’) is SS’ = SS/(1+SS/N). For this study, the population size (N) was 360, which is the number of patients admitted with acute ischemic stroke at RIMS, Ranchi, between 2019 and 2020. Using this information, the sample size (SS’) was calculated to be 185.

The inclusion criteria for this study were as follows: (1) patients aged more than 18 years; (2) individuals whose initial acute ischemic stroke episode was verified by radiological evidence, clinical examination, and medical history; and (3) patients or guardians who provided consent. The exclusion criteria were as follows: (1) patients with a hemorrhagic stroke; (2) patients using anticoagulants and antiplatelets or with pre-existing coagulopathy; (3) patients with a prior history of transient ischemic attack (TIA) or stroke; (4) patients with cardiac arrhythmia or valvular heart disease; (5) patients with any malignancies; and (6) patients with end-stage renal disease. In the control population, acute ischemic stroke was ruled out in addition to the above-mentioned exclusion criteria.

Informed written consent was taken from all patients/guardians and control patients who fulfilled the inclusion criteria. This study received approval from the Institutional Ethics Committee, RIMS, Ranchi (Memo No.: 286/IEC, RIMS; dated: 21/6/2021).

The data were gathered and documented in a specifically designed proforma. Blood pressure measurement, urea, creatinine, complete blood count, fasting lipid profile, random blood glucose level, and electrocardiogram were done for all patients. The HbA1c was determined using the ion-exchange high-performance liquid chromatography (HPLC) method. The same method was used for HbA1c measurements in the control patients.

All measurements were added to a Microsoft Excel sheet (Microsoft Corporation, Redmond, WA), and the data were analyzed using SPSS version 22.0 software (IBM Corp., Armonk, NY). After performing a descriptive statistical analysis, the results were classified over a range of values and described appropriately. For the comparison of each variable between the ischemic stroke cases and the controls, an independent t-test was performed. A multivariable logistic regression analysis was performed to select the potential variables and ascertain the associations using a multivariable analysis. A p-value less than or equal to 0.05 was regarded as statistically significant.

## Results

In total, 185 ischemic stroke patients and 185 control patients were included in this study. Out of the 185 ischemic stroke patients included in this study, 83 were females, while 102 were males. Of the 185 control patients, 85 were females, and 100 were males. The mean age of the ischemic stroke patients was 63.77 ± 10.312 years, and the mean age of the control population was 53.18 ± 11.35 years. The range of the age distribution in the ischemic stroke cases was 41-86.

The range of HbA1c levels in ischemic stroke patients was between 4.5 and 14.2%. Of the 185 ischemic stroke patients, 34 had an HbA1c level less than 5.7%, 68 had an HbA1c level between 5.7 and 6.4%, and 83 had an HbA1c level more than 6.4%. Of the 185 controls, 111 had an HbA1c level less than 5.7%, 40 had an HbA1c level between 5.7 and 6.4%, and 34 had an HbA1c level over 6.4%. Ischemic stroke patients had a mean HbA1c of 6.97 ± 1.84%, while the control population had a mean HbA1c of 5.99 ± 1.69% (Table 1).

**Table 1:** Mean HbA1c value of the cases and controls. HbA1c: glycated hemoglobin.

Parameter	Population	Mean value	Independent sample t-test p-value
HbA1c	Cases	6.97 ± 1.84	<0.01
	Controls	5.99 ± 1.69	

Male patients had a mean HbA1c of 7.05 ± 1.84%, and female patients had a mean HbA1c of 6.86 ± 1.84% (Table 2).

**Table 2:** HbA1c levels based on the sex of ischemic stroke patients. HbA1c: glycated hemoglobin.

Parameter	Population	Mean value	Independent sample t-test p-value
HbA1c	Males	7.05 ± 1.84	0.495
	Females	6.86 ± 1.84	

Of the 185 ischemic stroke patients, 78 had a random blood sugar (RBS) of less than 140 mg/dL, 58 had an RBS between 140 and 199 mg/dL, and 49 had an RBS of 200 mg/dL or higher. Of the 185 control patients, 121 had an RBS of less than 140 mg/dL, 33 had an RBS between 140 and 199 mg/dL, and 31 had an RBS of 200 mg/dL or more (Table 3). Ischemic stroke patients had a mean RBS of 170.21 ± 84.16 mg/dL, and control patients had a mean RBS of 150.03 ± 82.25 mg/dL (Table 4).

**Table 3:** Distribution of cases and controls based on RBS level. RBS: random blood sugar.

RBS values	Population	
	Cases	Controls
0-139	78	121
140-199	57	33
≥200	50	31

**Table 4:** Mean RBS level in cases and controls. RBS: random blood sugar.

Parameter	Population	Mean value (mg/dl)	Independent sample t-test p*-*value
RBS	Cases	170.21 ± 84.16	0.02
	Controls	150.03 ± 82.25	

The mean values for total cholesterol, high-density lipoprotein (HDL), low-density lipoprotein (LDL), and triglycerides in ischemic stroke patients were 156.52 ± 51.87, 42.51 ± 9.97, 85.98 ± 25.06, and 136.24 ± 55.53, respectively. The mean values for total cholesterol, HDL, LDL, and triglycerides in the controls were 150.63 ± 48.97, 46.16 ± 8.84, 84.77 ± 21.79, and 134.42 ± 61.69, respectively (Table 5).

**Table 5:** Descriptive statistics of different variables of lipid profile.

Variable	Population	Mean value	p-value
Total cholesterol	Cases	156.51 ± 51.87	0.26
	Controls	150.63 ± 48.97	
High-density lipoprotein	Cases	42.51 ± 9.97	<0.01
	Controls	46.16 ± 8.84	
Low-density lipoprotein	Cases	85.98 ± 25.06	0.62
	Controls	84.77 ± 21.79	
Triglycerides	Cases	136.24 ± 55.53	0.77
	Controls	134.42 ± 61.69	

Of the 185 ischemic stroke patients, 84 were hypertensive and 101 were non-hypertensive. Of the 185 control patients, only 64 were hypertensive (Table 6).

**Table 6:** Distribution of hypertensive status in cases and controls.

Blood pressure	Population		Pearson chi-square p-value
	Cases	Controls	
Hypertensive	84	64	0.034
Non-hypertensive	101	121	

To compare the factors previously determined to be statistically significant between the ischemic stroke cases and control patients, a multivariable logistic regression analysis was performed. The odds ratios of the different variables are listed in Table 7.

**Table 7:** The odds ratio of different variables. HbA1c: glycated hemoglobin; HTN: hypertension; HDL: high-density lipoprotein.

Variables	P-value	Odds ratio	95% CI
HbA1c	0.001	1.280	1.11 to 1.48
Age	<0.001	1.085	1.06 to 1.11
HTN	0.618	1.130	0.70 to 1.83
HDL	0.055	0.975	0.95 to 1.00

The data suggest that even after considering other variables, the differences in age and HbA1c between the ischemic stroke cases and control patients were statistically significant. However, neither HDL nor known hypertension states were statistically significant. Also, it was concluded that an increase in HbA1c by 1% increases the risk of ischemic stroke by 28% (odds ratio: 1.28; 95% CI: 1.11-1.48) (Table 7).

A logistic regression analysis was performed to compare the risk of cerebral ischemic stroke in prediabetic and diabetic patients. For this analysis, HbA1c levels of less than 5.7% were used as the reference group.

It was concluded that the population with an HbA1c level over 6.4% (odds ratio: 5.55; 95% CI: 3.21 to 9.60) have a greater risk of ischemic stroke than those with an HbA1c level between 5.7 and 6.4% (odds ratio: 7.97; 95% CI: 4.58 to 13.87) (Table 8).

**Table 8:** Odds ratio based on HbA1c values after considering confounders. HbA1c: glycated hemoglobin.

HbA1c level	p-value	Odds ratio	95% CI
5.7-6.4	<0.001	5.14	2.78 to 9.50
>6.4	<0.001	5.50	2.99 to 10.14

**Figure 1:**
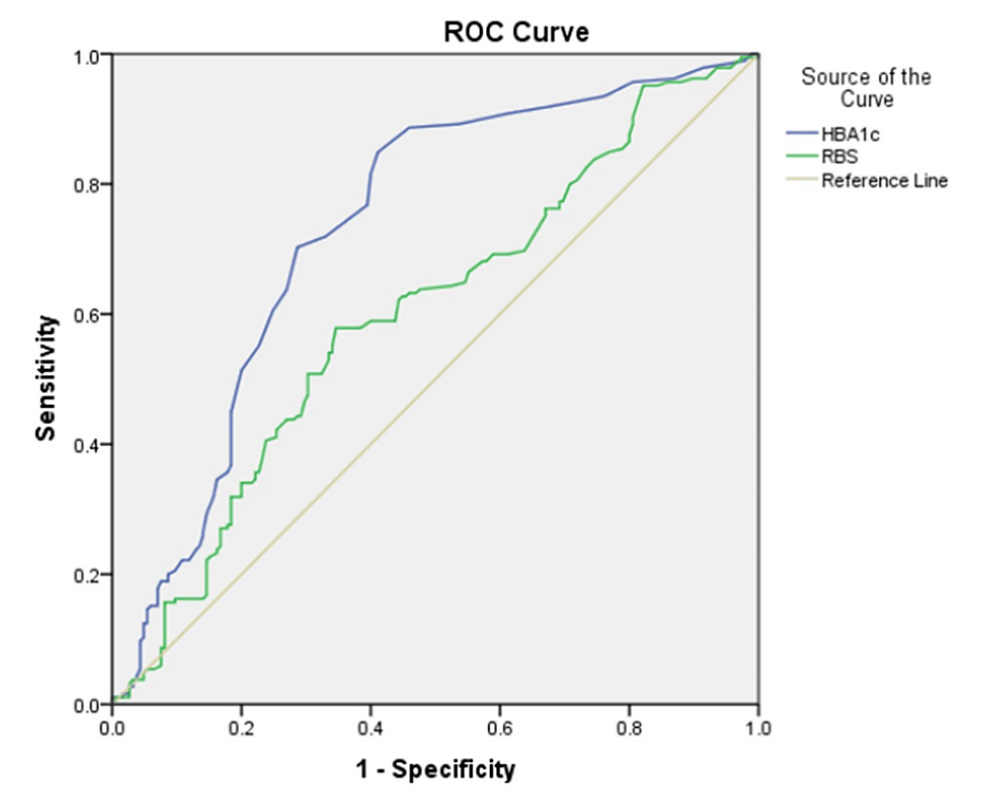
Logistic ROC analysis for HbA1c and RBS. ROC curve: receiver operating characteristic curve; HbA1c: glycated hemoglobin: RBS: random blood sugar.

A multivariable logistic regression analysis was performed to compare the variables previously determined to be statistically significant between the ischemic stroke cases and control patients. From the analysis, it was concluded that RBS was not statistically significant between the two groups when other confounders were taken into consideration (Table 9). To compare the HbA1c and RBS of patients, a receiver operating characteristic (ROC) curve was made (Figure 1).

**Table 9:** The odds ratio based on RBS levels after considering confounders. RBS: random blood sugar; HTN: hypertension; HDL: high-density lipoprotein.

Variables	P-value	Odds ratio	95% CI
RBS	0.592	1.001	0.998 to 1.004
Age	<0.001	1.090	1.064 to 1.116
HTN	0.719	1.091	0.680 to 1.748
HDL	0.014	0.970	0.946 to 0.994

The area under the curve (AUC) for HbA1c was 0.733 and the AUC for RBS was 0.600. This suggests that HbA1c is a superior marker to RBS in accurately assessing glycemic control in acute ischemic stroke patients. Hence patients with increased HbA1c have a greater risk of ischemic stroke compared with patients with a high RBS. It is also concluded that the risk of ischemic stroke increases significantly with HbA1c levels above the threshold of 5.9%.

## Discussion

We observed that HbA1c was significantly elevated in ischemic stroke patients compared with the controls after adjusting for confounding variables and age-adjusted risks. As HbA1c increased, the odds ratio for ischemic stroke also increased, with the risk of ischemic stroke nearly seven times higher in those with an HbA1c over 6.4% than in those with an HbA1c under 5.7%. There was a discernible upward trend in the chances of having an ischemic stroke as the HbA1c level increased, particularly in patients with an HbA1c above 5.9%, and this relationship was nearly constant. In this study, the significance of the HbA1c level was more pronounced than the RBS level. HbA1c is a better marker than RBS in accurately assessing glycemic control. This implies that long-term exposure to elevated blood glucose levels may contribute to the development of a stroke. This study also reports that there was a 28% increase in the associated risk of a stroke for every 1% increase in HbA1c after adjusting for confounding variables and age-adjusted risks.

A study by Selvin et al. monitored a community-based non-diabetic adult population for 14 years, and individuals with HbA1c levels between 5.0% and 5.5% at the start of the study were compared with individuals with increased HbA1c values. The study found that as the baseline HbA1c levels increased, their risk of ischemic stroke also increased linearly. These results were more significant for HbA1c than for fasting plasma glucose [12]. Chang et al., in a retrospective cohort study, concluded that the risk of having another stroke was higher in individuals with higher HbA1c levels (6.8-7.0%) [13]. The European Prospective Investigation into Cancer/Norfolk study carried out in the United Kingdom studied the association between HbA1c and the incidence of stroke in 10,489 patients lacking any diagnosed diabetes and stated that only patients with an HbA1c over 7.0%, who were probably individuals with undiagnosed diabetes, had a significantly higher risk of stroke indicating the presence of a threshold [14]. Watanabe et al. reported that an elevation in HbA1c levels was associated with a higher risk of ischemic stroke and was especially higher in patients with an HbA1c of 6.5% or higher [15]. Nomani et al. concluded that an elevated risk of ischemic stroke was indicated by an HbA1c higher than 5.6% [16]. Studies by Oh et al., Ahmed et al., and Raj et al. reported that increased HbA1c showed a markedly significant increase in ischemic stroke after adjusting for confounding variables [17-19]. These results align with this present study in which a continuous correlation between the HbA1c values and the risk of ischemic stroke was observed. However, the threshold at which the association becomes statistically significant exhibits inconsistent findings across various studies.

In the United States of America, the Atherosclerosis Risk in Communities Study investigated the relationship between HbA1c and ischemic stroke in nearly 10,000 non-diabetic individuals and nearly 1,600 diabetics and concluded that the incidence of ischemic stroke increased constantly with the rise in HbA1c level, starting with the 6.8% HbA1c category, indicating the lack of a threshold [12]. It has been demonstrated that having diabetes raises the risk of stroke; however, it has not been specifically suggested whether the risk of stroke is correlated with blood glucose levels below the diagnostic threshold for diabetes [20]. In a large cohort of 2,37,468 people, the Asia Pacific Cohort Studies Collaboration recently investigated the correlation between fasting blood glucose levels and the incidence of stroke and found a linear association between the incidence of stroke and fasting blood glucose levels [21]. This current study discovered that the risk of an ischemic stroke did not substantially correlate with an increase in RBS levels after adjusting for confounding variables and age-adjusted risks. This may be because the random blood glucose level is not a reliable indicator of chronic hyperglycemia due to its considerable intraindividual variation. Myint et al. concluded that the relative risks (95% CI) of a stroke for participants with HbA1c of 5-5.4%, 5.5-6.9%, and 7% or higher were 0.78, 0.83, and 2.83, respectively, relative to individuals with an HbA1c less than 5% [14]. In this current study, the odds ratios (95% CI) of a stroke for participants with HbA1c levels of 5.7-6.4% and greater than 6.4% were 5.14 and 5.50, respectively, compared with those with an HbA1c of less than 5.7%. The variable results could be due to a smaller sample size in the current study and hospital-based bias. Patients with ischemic stroke can have variable RBS levels due to dextrose infusion, stress hyperglycemia, and poor food intake. Thus, HbA1c levels should be routinely tested in individuals with a risk of ischemic stroke.

This study had a few limitations: (1) only a single HbA1c level was taken. This indicates glycemia for three months and undervalues any long-term association. (2) HbA1c was compared with RBS levels to determine the risk of ischemic stroke, and an oral glucose tolerance test (OGTT) could not be done. HbA1c combined with an OGTT would have a higher sensitivity and specificity for assessing the risk of ischemic stroke. (3) This was a single-center case-control study, and thus caution should be exercised in assuming a precise relationship between HbA1c and ischemic stroke. (4) Individuals taking antidiabetic medications at the baseline could not be excluded. The relationship between HbA1c and stroke risk may have been underestimated because some individuals whose HbA1c had been lowered with medication were included. (5) When calculating the odds ratio, family history of cardiovascular and cerebrovascular disease, BMI, and smoking were not considered. (6) This study was conducted in a tertiary care hospital so hospital bias cannot be completely ruled out.

## Conclusions

This study shows that patients with elevated HbA1c are at a greater risk of ischemic stroke. In addition, RBS levels should not be relied on to rule out diabetes in ischemic stroke patients. HbA1c was found to be a superior predictor of assessing glycemic control than RBS levels, and thus, HbA1c should be checked routinely in patients at risk of an ischemic stroke. The findings of this study indicate that HbA1c can be utilized as a marker to predict the risk of ischemic stroke.

## Appendices

**Table 10:** Proforma. Y: yes; N: no; HTN: hypertension; BP: blood pressure; RR: respiratory rate; CVS: cardiovascular system; CNS: central nervous system; INR: international normalized ratio; APTT: activated partial thromboplastin time; CBC: complete blood count; NCCT: non-contrast computed tomography.

Serial No.:	1	2	3	4	5	6	7	…	185
Name									
Age									
Sex									
ID No.									
Date of admission									
Chief complaint									
Comorbid status: Diabetes (Y/N), HTN (Y/N), coagulopathy (Y/N), valvular disease (Y/N), malignancy (Y/N), renal failure (Y/N)									
General examination: Pallor, icterus, cyanosis, clubbing, lymphadenopathy									
Vitals: Pulse, BP, RR, temperature									
Systemic examination: Respiratory, CVS, per abdomen, CNS									
Investigations: Prothrombin time, INR, APTT, CBC, blood urea, serum creatinine, fasting lipid profile, random blood glucose, 2D echo, carotid artery Doppler, NCCT brain, other relevant investigation									
Final diagnosis									
